# Development and Validation of a Rapid Assessment Version of the Assessment Survey of Primary Care in China

**DOI:** 10.3389/fpubh.2022.852730

**Published:** 2022-05-10

**Authors:** Chenwen Zhong, Junjie Huang, Lina Li, Zhuojun Luo, Cuiying Liang, Mengping Zhou, Li Kuang

**Affiliations:** ^1^Department of Health Policy and Management, School of Public Health, Sun Yat-sen University, Guangzhou, China; ^2^Jockey Club School of Public Health and Primary Care, The Chinese University of Hong Kong, Hong Kong, China; ^3^Bureau of Veteran Cadres of the Huadu District Party Committee, Guangdong, China

**Keywords:** COSMIN checklist, confirmatory factor analysis, exploratory factor analysis, primary care, validation

## Abstract

**Background:**

Measuring quality of primary care has attracted much attention around the world. Our team has developed and validated an Assessment Survey of Primary Care (ASPC) for evaluating quality of primary care in China. To facilitate the daily use of ASPC, this study aimed to develop and validate a rapid assessment version of ASPC (RA-ASPC) in China.

**Methods:**

This is a multi-phase study on 21 experts and 1,184 patients from 12 primary care facilities in 10 cities in China. Importance, representativeness, easy understanding, and general applicability of each item in ASPC scale were rated to select the top two ranked items for constituting RA-ASPC. Reliability of RA-ASPC was tested by calculating both Cronbach's alpha and McDonald's omega coefficients. Structural validity was assessed by exploratory and confirmatory factor analysis (EFA and CFA). Concurrent validity was performed by analyzing the relationship between RA-ASPC and patient satisfaction. Discriminant validity was tested by assessing the difference of RA-ASPC scores between patients with or without family doctors.

**Results:**

Ten items were selected for RA-ASPC. Both Cronbach's alpha (0.732) and McDonald's omega (0.729) suggested satisfactory internal consistency. In EFA, explained variance of RA-ASPC (72.6%) indicated its ability to measure quality of primary care in China. CFA indicators showed convincing goodness-of-fit (GFI = 0.996, AGFI = 0.992, CFI = 1.000, NFI = 0.980, RMR = 0.022, and the RMSEA = 0.000) for RA-ASPC. Positive association between RA-ASPC and patient satisfaction supported the concurrent validity of RA-ASPC. Patients with family doctors perceived higher quality of primary care than those without family doctors, indicating good discriminant validity of RA-ASPC.

**Conclusion:**

The theoretical framework of RA-ASPC was in line with internationally recognized core functions of primary care. Good psychometric properties of RA-ASPC proved its appropriateness in assessing quality of primary care from patients' perspectives in China.

## Introduction

Primary care is considered a regular entry point into health systems and central to improving accountability in health service delivery (1). Countries with optimal primary care reported lower health expenditure, fewer hospital admissions, and better clinical outcomes (1, 2). In 2018, WHO highlighted that concept misunderstanding and inappropriate methods were important challenges to improving quality of primary care (3). To strengthen primary care, clear and explicit measures for assessing the process quality of primary care are of significance (4, 5). There are existing international tools developed in western countries with a long history of primary care, such as the General Practice Assessment Questionnaire (GPAQ) developed in the UK (6), the Primary Care Assessment Tools (PCAT) developed in the US (7), and the EUROPE tool developed in Europe (8).

Being the largest and most populous developing country in the world, an extensive and massive primary care system has been developing rapidly in China (9), with an aim to provide high-quality primary care services to all inhabitants in the near future (10). To monitor the progress of the development process, as well as evaluate the construction and performance of primary care settings, a local-applicable evaluation tool should be developed to measure quality of primary care in China. Attempts have been made to apply the above-mentioned tools to evaluate quality of primary care in China (11–13). Results showed that the reliability and validity of these tools were barely acceptable, but they can be hard to achieve cross-culture equivalence in China, mainly due to the variations in population and language habits (14). In this case, new scales appropriate to measure quality of primary care in Chinese primary care practice are mandated.

Our team have previously developed and validated the Assessment Survey of Primary Care (ASPC) scale, to assess the quality of primary care in China (15, 16). The original ASPC scale was proved to be acceptable and user-friendly by patients, GPs, and leaders of primary care settings. With 41 items included in the original ASPC scale, ~10–15 min should be taken for completing the original ASPC scale. A long questionnaire may damage the adoption and scaling-up to a larger population. To achieve a better response rate and optimize the implementation of the scale in real-world practice, it is necessary to develop a rapid assessment version of ASPC scale (RA-ASPC). This study aimed to develop and validate an RA-ASPC using a multi-phase approach, including Phase I item development; and Phase II scale validation according to the COnsesus-based Standards for the selection of health Measurements Instruments (COSMIN) checklist (17).

## Methods

### Phase I Development of RA-ASPC Items

In Phase I, we assessed and selected items from the original ASPC scale to form RA-ASPC. The importance of data such as item loading is well-recognized in developing and validating a scale, including ASPC. However, items with good factor loadings may not be considered as representative or appropriate to the targeted construct and assessment objectives of RA-ASPC from experts' perspectives. Evaluation by experts who are highly knowledgeable about the domain of interest and scale development is significant for researchers to ensure that the hypothesis elaborated in the research appropriately represents the construct of interest (18). In this study, eligible experts were academic researchers with rich experience in primary care research, general practitioners with pragmatic clinical expertise in primary care, and frontline investigators who were most familiar with patient's experience in primary care. They were identified to provide unique and diverse insights on whether the item could be selected to constitute the domain by assessing content relevance, representativeness, and technical quality of the scale (18). Purposive sampling is a technique widely used for identifying individuals who are especially knowledgeable about, or experienced with, a phenomenon of interest (19). In this study, purposive sampling was adopted to recruit potential eligible experts. It also allows selection of experts who are willing to invest time and effort in the development process. All experts were approached through an author's professional network (LK). Experts included in this study were de-identified to safeguard confidentiality and independence of judgements during the whole process.

After collecting verbal informed consent from eligible experts who agreed to participate, experts were then invited to make judgements on items in each domain of original ASPC scale based on the following criteria (20, 21): (i) perceived importance, referring to the importance level of the item to the embedded domain; (ii) representativeness, measuring the extent to which the items captured the relevant experience of the patients and represented the embedded domain; (iii) easy understanding, testing for item difficulty by asking how easy/difficult it is for patients to understand the item concerning their cognitive level; and (iv) general applicability, referring to whether the item could be generally applied to different primary care practices. Experts' judgements of perceived importance, representativeness, easy understanding, and general applicability on each item were presented in percentage (%). An average percentage of the four criteria was calculated to represent the score of recommendation from experts' perspectives. Two items with the highest score of recommendation were selected to constitute the five domains (first-contact care, continuity, accessibility, comprehensiveness, and coordination) in RA-ASPC.

### Phase II Validation of RA-ASPC

In Phase II, we assessed both reliability and validity of RC-ASPC following the guidance of COSMIN checklist (17). Reliability of the scale was evaluated by two indicators: Cronbach's Alpha (22) and McDonald's Omega (23). The adoption of two indicators aimed to increase the reliability of the interpretation, since limitations such as reliability inconsistencies have occurred through Cronbach's Alpha (24, 25). Meanwhile, reliability of all the five domains was also assessed using the Spearman-Brown coefficient as these domains were comprised of two items (26). A coefficient value >0.7 was regarded as satisfactory internal consistency (27). Coefficient within 0.5–0.7 was considered to represent an acceptable level of internal consistency (28, 29). Content validity was measured by expert's assessment on each item constituted the domain for perceived importance, representativeness, easy-understanding, and general applicability as stated in the previous section (30, 31). Structural validity was measured by employing both exploratory factor analysis (EFA) and confirmatory factor analysis (CFA) (17). Concurrent validity (17) was assessed by examining the relationship between RA-ASPC and patient satisfaction. Discriminant validity (32) was demonstrated by significant differences in quality of primary care assessed by the RA-ASPC between patients with or without a family doctor. Detailed information was reported as follows.

The EFA was conducted on a randomly selected sample of 581 patients from the whole sample (1,185 patients) using Principal Components Analysis and varimax rotation method. Bartlett's test of sphericity at a significance level (*p* < 0.05) and a high value of the Kaiser–Meyer–Olkin (KMO). Measure of Sampling Adequacy (>0.6) would indicate sample adequacy for factor analysis (33, 34). Explanatory variance of the scale should be around 60% (35). Based on the previous theoretical framework, a total of five factors were predefined to be extracted. Items with loadings higher than 0.4 were considered acceptable (36).

After the factor structure was derived from EFA, confirmatory factor analysis (CFA) was conducted to test how well the model fits the data using Diagonally Weighted Least Squares (DWLS) estimation method (37). To conduct CFA, the remained sample (*N* = 605) were used. A model could be considered as an adequate fit to the data once the following criteria were met (38–40): (i) the chi-square goodness of fit test (χ^2^/df) <3; (ii) Goodness of Fit Index (GFI)>0.90; (iii) Comparative fit index (CFI)>0.90; (iv) the Normed Fit Index (NFI)>0.85; and (v) Root Square Means Error of Approximation (RSMEA) <0.05. CFA was conducted using the “lavaan” package in R version 4.1.2 (41).

For concurrent validity, the relationship between scores of five RA-ASPC domains and patient satisfaction with the GP was assessed. Patient satisfaction was chosen as it was an important indicator for assessing the performance of primary care (42). A linear regression model was performed to explore the relationship between the score of RA-ASPC and patient satisfaction, after controlling for patient's sociodemographic information and health care utilization.

To assess discriminant validity, RA-ASPC scores were compared between patients with or without family doctors. According to previous studies, patients who had a contracted GP tend to experience a higher quality of primary care (43, 44). Therefore, we expected that patients with a family doctor have higher RA-ASPC scores compared with patients without a family doctor. Multivariate analyses of covariances (ANCOVAs) were conducted to compare scores of the RA-ASPC domains between the two groups of patients after adjusted by patient sociodemographic information and health care utilization. A *P* < 0.05 was considered statistically significant. The EFA, concurrent validity, and discriminant validity were conducted by IBM SPSS 26.0.

### Settings, Participants and Procedures

Data were collected from 12 primary care settings (10 community health centers in urban areas and two township health centers in rural areas) in 10 cities of Guangdong, China from Jan to Apr 2019. Eligible patients were those who were aged 18 years or older, could speak Mandarin or Cantonese, and had visited the current primary care settings three times or more. Patients with mental disorders were excluded. Eligible patients were invited to participate in our study with a full explanation of research purpose of this study and were told that it would not influence their GP visits. After patients agreed to participate in this study, verbal informed consent was obtained. Individual face-to-face and one-to-one interviews were then conducted in the waiting area. A total number of 1,185 patients was recruited. After the interview process, all questionnaires were reviewed and checked right to ensure no missing data.

In the patient questionnaire, there are four main sections including sociodemographic characteristics, healthcare utilization in the primary care settings, RA-ASPC items, and satisfaction with their GPs. Patients were asked to rate each item based on their previous experience of primary care in the primary care setting, but not based on their expectation of services. Patient satisfaction was assessed by asking patients to rate their overall satisfaction level with the GPs from 1 to 100. Items in RA-ASPC were assessed using a 4-point Likert-type scale (1 = never; 2 = sometimes; 3 = often; 4 = always). An average score of the two items for each domain was considered as the score of the respective domain. An average score of the five domains was calculated to summarize the overall quality of primary care.

### Ethical Approval

Ethical approval was obtained from the Institutional Review Board of School of Public Health, Sun Yat-Sen University, P.R. China (2018.014). The protocol for this study conforms to the principles embodied in the Declaration of Helsinki.

## Results

### Participants

A total of 21 experts were included. Among them, five experts were academic researchers with rich experience in primary care research, seven experts were clinical professionals working in primary care settings, and nine experts were frontline investigators who were most familiar with patients experience of primary care. For patients included in this study, most patients were female (61.9%), married (85.7%), with an education level of middle/high school (54.4%), employed (48.8%), and with a monthly household income less than CNY 5000 (74.9%). In terms of health status, most (86.8%) patients stated their health as “general” or “good”. More than half of patients had connections with the primary care settings for 5 years, indicating that they have rich experience in the primary care settings. In addition, 63% of patients had a family doctor. Detailed information of the participants could be found in Tables 1, 2.

**Table 1:** Socio-demographic characteristics of the experts participated in selecting items for RA-ASPC (*N* = 21).

	**N**	**%**
Gender		
Male	8	38.1
Female	13	61.9
Age (years) (mean ± SD)	31.4 ± 4.85
Education level		
Bachelor's degree	7	33.3
Master's degree	12	57.1
PhD degree	2	9.5
Work experience in primary care settings (years)		
<5	11	52.4
5–10	4	19.0
11–15	5	23.8
16–20	1	4.8
Professional		
Academic researchers	5	23.8
General practitioners	7	33.3
Frontline investigators	9	42.9

*SD, standard deviation; ASPC, Assessment Survey of Primary Care; RA-ASPC, Rapid assessment of the ASPC scale*.

**Table 2:** Characteristics of patients included in this study: *N* (%).

**Patient characteristics**	**Random sample**	**Random sample**	**Total**
	**for EFA**	**for CFA**	**(*N* = 1,185)**
	**(*N* = 581)**	**(*N* = 604)**	
Gender			
Male	243 (41.8)	208 (34.4)	451 (38.1)
Female	338 (58.2)	396 (65.6)	734 (61.9)
Age (years)			
<45	225 (38.7)	246 (40.7)	471 (39.7)
45–65	217 (37.3)	205 (33.9)	422 (35.6)
>65	139 (23.9)	153 (25.3)	292 (24.6)
Marital status			
Unmarried	43 (7.4)	38 (6.3)	81 (6.8)
Married	496 (85.4)	521 (86.3)	1,017 (85.8)
Divorced or widowed	42 (7.1)	45 (7.5)	87 (7.3)
Education			
Primary school or below	165 (28.4)	149 (24.7)	314 (26.5)
Middle/high school	314 (54.0)	331 (54.8)	645 (54.4)
Bachelor's degree or above	102 (17.6)	124 (20.5)	226 (19.1)
Employment status			
Employed	289 (49.7)	289 (47.8)	578 (48.8)
Retired	141 (24.3)	154 (25.5)	295 (24.9)
Unemployed	151 (26.0)	161 (26.7)	312 (26.3)
Monthly household Income (CNY)			
≤ 5,000	443 (76.2)	445(73.7)	888 (74.9)
5,001–10,000	78 (13.4)	96(15.9)	174 (14.7)
>10,000	60 (10.3)	63(10.4)	123 (10.4)
Household status			
Local residence	330 (56.8)	352 (58.3)	682 (57.6)
Non-local residence	251 (43.2)	252 (41.7)	503 (42.4)
Health insurance			
Urban employee basic medical insurance	218 (37.5)	227 (37.6)	445 (37.6)
Urban residence basic medical insurance	51 (8.8)	58 (9.6)	109 (9.2)
Basic medical insurance	102 (17.6)	130 (21.5)	232 (19.6)
Other insurances	141 (24.3)	128 (21.2)	269 (22.7)
Without medical insurance	69 (11.9)	61 (10.1)	130 (11)
Health status			
Poor	80 (13.8)	77 (12.7)	157 (13.2)
General	260 (44.8)	279 (46.2)	539 (45.5)
Good	241 (41.5)	248 (41.1)	489 (41.3)
With or without chronic diseases			
Yes	265 (45.6)	271 (44.9)	536 (45.2)
No	316 (54.4)	333 (55.1)	649 (54.8)
Number of years since first visit to the primary care setting			
<2 year	129 (22.2)	144 (28.8)	273 (23.0)
2–5 years	139 (23.9)	147 (24.3)	286 (24.1)
More than 5 years	313 (53.9)	313 (51.8)	626 (52.8)
Whether have a family doctor			
Yes	377 (64.9)	370 (61.3)	747 (63)
No	204 (35.1)	234 (38.7)	438 (37)

### Results of Phase I Development of RA-ASPC

**Figure 1:**
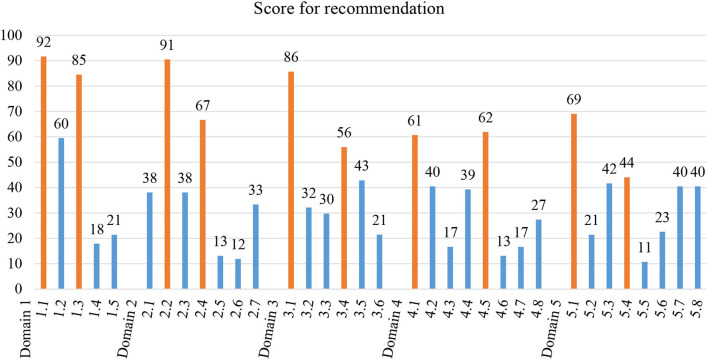
Item selection after expert's consensus based on the score for recommendation, presented as the average percentage of the four criteria. The top two items with the highest average percentage were considered to reach expert's consensus and selected to constitute the rapid assessment of the ASPC scale, which were highlighted in orange. Details of the items and domains could be found in Appendix 1.

The average score of recommendations for each item in each domain is presented in Figure 1. Detailed results of experts' ratings for each item of the four criteria could be found in Appendix 1. The full range of the 0-to-100 score distribution was observed for all domains. The mean scores of items ranged 18–92 for first-contact care, 12–91 for continuity, 21–86 for accessibility, 13–62 for comprehensiveness, and 11–69 for coordination. The top two ranked items in each domain were selected to form RA-ASPC accordingly. Detailed definitions of each item constituted the five domains of RA-ASPC are presented in Appendix 2.

### Results of Phase II Reliability and Validity of RA-ASPC

Items mean score of RA-ASPC ranged from 2.64 ± 0.87 to 3.87 ± 0.47 (Table 3). All selected items fulfilled experts' judgements, so content validity was claimed in this case. Results of the reliability are shown in Table 3. Cronbach's alpha coefficient for RA-ASPC was 0.732, while McDonald's omega value was 0.729, indicating a good level of internal consistency. For the five domains of RA-ASPC, Spearman-Brown coefficients of four domains ranged between 0.526 and 0.777, indicating an acceptable to a good level of internal consistency. Except for accessibility, the coefficient was 0.441. Removal of any items may cause a decrease of the scale Cronbach's alpha.

**Table 3:** Item analysis and factor analysis of the RA-ASPC (*N* = 581).

**Item contents**	**Mean ±SD**	Factor loadings^*^					**Communality**	**Explained variance (%)**	**Cronbach's α**	**Spearman-Brown coefficients**	**Scale Cronbach's α if Item Deleted**	**McDonald's Omega**
		**1**	**2**	**3**	**4**	**5**						
Factor 1 Coordination								16.9	0.750	0.777		
Item 5.1	2.64 ± 0.87	0.862					0.791				0.701	
Item 5.4	2.81 ± 0.62	0.837					0.793				0.698	
Factor 2 First-contact care								15.6	0.705	0.707		
Item1.1	3.50 ± 0.93		0.895				0.817				0.716	
Item1.3	3.23 ± 1.03		0.784				0.738				0.694	
Factor 3 Comprehensiveness								14.9	0.525	0.526		
Item 4.1	2.86 ± 1.12			0.827			0.717				0.713	
Item 4.5	2.71 ± 1.18			0.733			0.622				0.706	
Factor 4 Continuity								13.4	0.555	0.555		
Item2.2	2.73 ± 1.01				0.900		0.839				0.721	
Item2.4	2.76 ± 1.03				0.640		0.654				0.690	
Factor 5 Accessibility								12.9	0.376	0.441		
Item 3.1	3.87 ± 0.47					0.811	0.728				0.730	
Item 3.4	3.16 ± 0.91					0.753	0.683				0.730	
RA-ASPC								72.6	0.732			0.729
KMO = 0.74, Bartlett's test: χ2 = 1,177.0 (*p* < 0.001)												

*SD, Standard Deviation; CI, Confidence Interval; KMO, Kaiser-Meyer-Olkin Measure of Sampling Adequacy. RA-ASPC, Rapid assessment of the ASPC scale*.

* *Rotation Method: Varimax with Kaiser Normalization. Details of the items could be found in Appendix 2*.

In addition, structural validity, concurrent validity, and discriminant validity of RA-ASPC were also confirmed. For EFA, the result of Bartlett's test of sphericity was 1177.0 (df = 45, *p* < 0.001) and the KMO for this sample was 0.74, indicating the data were appropriate for conducting factor analysis. The factor pattern and factor structure coefficients are presented in Table 3, along with commonalities of the measured variables. All 10 items had communalities ranging from 0.622 to 0.839. The proportion of total explained variance was 72.6%. Factor loadings for all items in the scale ranged from 0.640 to 0.900. In summary, all items' validity and quality in each domain were classified as excellent and very good.

**Figure 2:**
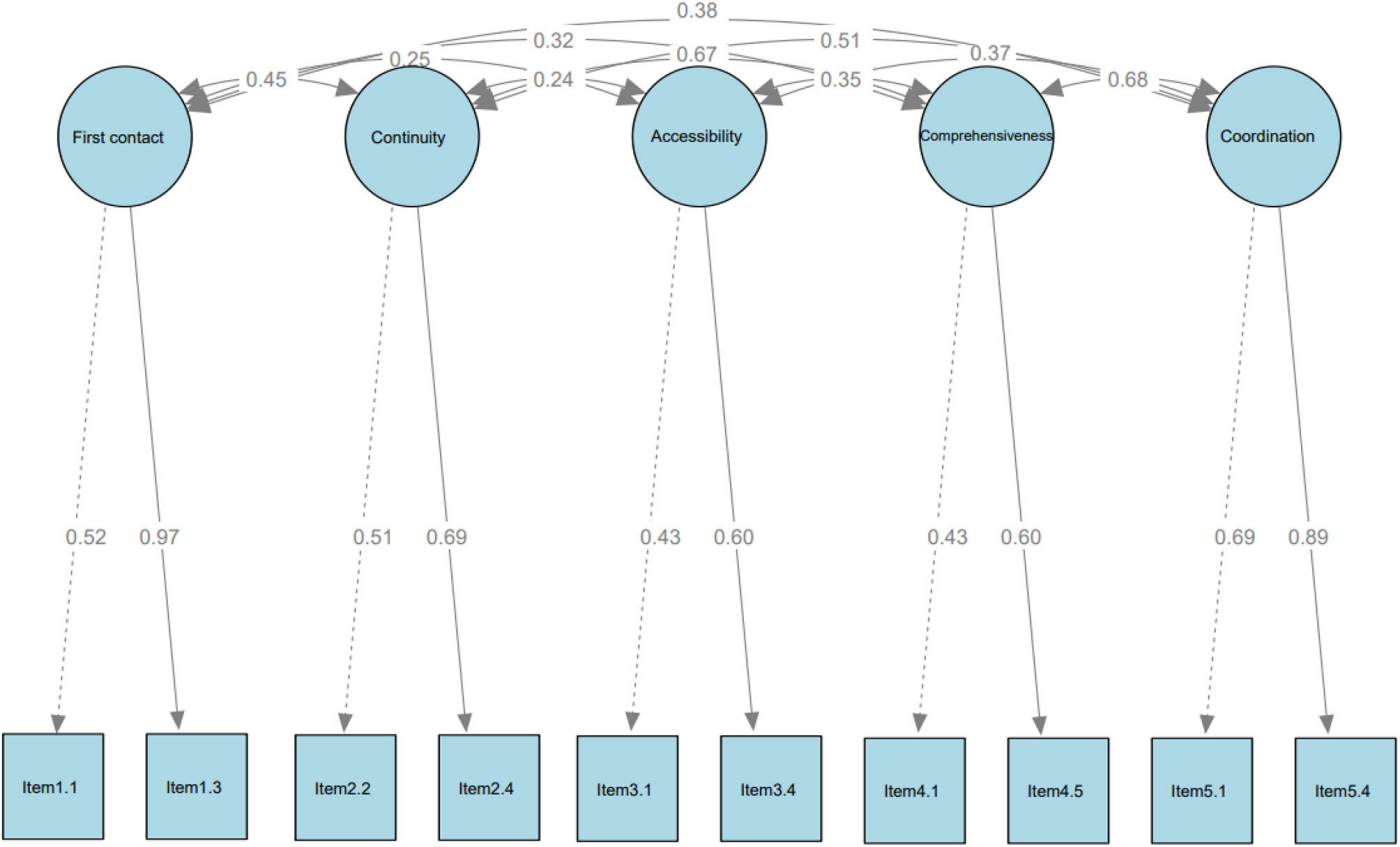
Confirmatory factor analysis of the RA-ASPC using Dampened weighted least squares (DWLS) as estimator (*N* = 604). Chi-square minimum = 24.081; Degree of freedom = 25; Goodness of Fit Index (GFI) = 0.996; Comparative Fit Index (CFI) = 1.000; Root Mean square Residual (RMR) =0.022; Root Mean Square Error of Approximation (RMSEA) = 0.000. Details of the items could be found in Appendix 2.

For CFA, the estimated path diagram with the standardized path coefficients, as well as the correlations coefficients between the latent variables for the hypothesized five-domain model are presented in Figure 2. The standardized path coefficients were all statistically significant. All fit indices (χ^2^ = 24.08, df = 25, GFI = 0.996, AGFI = 0.992, CFI = 1.000, NFI = 0.980, RMR = 0.022, and the RMSEA = 0.000) showed a good model fit with the data used in the present study. In CFA, the same structure of 10 items distributed across five factors was kept, in agreement with the expert's perception and exploratory validation. The results confirmed the validity of RA-ASPC by showing the conceptual suitability of the structure and reasonable fit.

Relationships between the score in each domain of RA-ASPC and patient satisfaction with the GP are shown in Appendix 3. The direction of the relationships supported the hypothesis that higher levels of quality of primary care were associated with a better achievement of patient satisfaction (β > 0, *P* < 0.05). Considering that, we could say that our scale has good concurrent validity. Results for discriminant validity by assessing the difference of RA-ASPC scores between patients with and without family doctors could be found in Appendix 4. We observed that there were statistically significant differences between the two groups, except for the domain of accessibility. However, the group of patients with family doctors still had higher scores in accessibility compared with patients without family doctors. These differences indicated that the scale had good discriminant validity.

## Discussion

This study aimed to develop and validate an RA-ASPC for assessing quality of primary care in China. Using a multi-phase approach, RA-ASPC was developed and validated on the basis of the original ASPC version. In Phase I, 10 items, which were most important, representative of the embedded domain, easily understood by patients, and universally applicable, were selected to form the RA-ASPC scale by experts. Following the guidance of the COSMIN checklist, psychometric properties of RA-ASPC were appraised comprehensively. Results of the validation procedures indicated good content validity via expert assessments, satisfactory reliability *via* both Cronbach's alpha and McDonald's omega coefficients, acceptable structural validity, as well as good concurrent validity and discriminant validity.

The RA-ASPC was derived from the original ASPC scale. All advantages of developing the original ASPC scale were also reflected in the RA-ASPC. In general, the development of the original ASPC scale was in charge of two important tasks. First, the original ASPC scale should have the ability to compare with existing literature and become one of the international scales for assessing quality of primary care in developing areas. Second, as a newly developed primary care assessment tool in China, it should be promising to be adopted in the local primary care practice, and feasible for the local health system structure and social context.

To accomplish the first task of the original ASPC scale, our team first constructed a theoretical framework with key components of the core functions of primary care (first-contact care, continuity, accessibility, comprehensiveness, and coordination) (45–47). Operational definitions and related items were established for the key components in each domain based on the following three principles (46, 47). The first principle was enriching the embedded components of each domain as comprehensively as possible (Exhaustion), to make sure the components adequately covered each domain. The second principle was avoiding overlaps of components and specifying boundaries of the domains (Mutual exclusion). The third principle was excluding any system factors that could hardly be modified, such as any system factors related to health financing and resources allocation by primary care facility itself (Controllability). For example, when we considered accessibility, we excluded geographical accessibility, and financing accessibility in this domain as these factors were barely changed. Focusing on factors that could be modified would make the evaluation more valuable to health providers and policy makers.

To accomplish the second task of the original ASPC scale, we then tailored each item to the current primary care practice in China. Based on the connotation of the aforementioned theoretical framework (45–47), we iteratively modified the item expression until it met the social cultural and vocabulary cognitive characteristics of Chinese residents. The following four-step approach was used to develop the original ASPC scale, including comprehensive literature search, expert focus groups, patient interviews, and a cross-sectional survey. Development and validation of the original ASPC scale was also conducted according to the COSMIN checklist (17).

As with the use of all brief versions of the original scales, the use of a rapid assessment version rather than the original scale is inevitably associated with the loss of a substantial amount of information. The scope and emphasis of RA-ASPC are to include items that best reflect the performance of current primary care practice in China, and meanwhile to reduce time required to answer the questionnaire, so as to facilitate the adoption at scale in real-world practice. For the original ASPC, it was designed to capture the richness and comprehensiveness of the core functions of primary care. The trade-offs of using RA-ASPC or the original one to measure quality of primary care should be made by instrument users or policymakers before adoption.

Regarding the subscales included in the RA-ASPC, in line with the well-described, universal functions, we measured five core functions of primary care in the RA-ASPC, including first-contact care, continuity, accessibility, comprehensiveness, and coordination (48). First contact care refers to the use of services for any new problem or new episode of a problem. Continuity refers to the longitudinal relationship between patients and their regular sources of care. Accessibility implies the extent to which patients can access primary care. Comprehensiveness indicates the availability of a wide range of services arranged to fulfill patients and populations functional, organic, or social needs. Coordination refers to the linkage to ensure a smooth transition between different levels of care for patients with complex health needs (15, 48). For patient-centeredness, the sixth domain included in the original ASPC scale, considering the scope of developing RA-ASPC, we did not include it in this version of scale. Firstly, patient-centered care could be delivered by adopting a person-centered approach in dealing with patients and their questions, such as using the triad of ideas, concerns, expectations (ICE) (49) or the BATHE consultation technique (50) in general practice consultations. In current practice, GP consultation was relatively short and emphasized on therapeutic issues (51), with less focus on the abovementioned patient-centered approach. Nevertheless, patient-centeredness can be interconnected through ubiquitous access to the usual source of trusted and competent care for most common conditions, as well as appropriate uncertainty management over time based on relationships and an understanding of patients within their context and community (48), which has already been measured in RA-ASPC.

### Comparison With Existing Literature

In Phase I, four indicators were adopted as criteria to ensure that items included in RA-ASPC were clear, simple, accurate and had high representativeness of patient's real experience on the current primary care practice in China. For example, in the domain of continuity, “GP's familiarity with patient's medical history” were selected rather than “GP's familiarity with patient's family members' medical history.” It reflected that more attention was paid to patient's medical history in the primary care practice of China at the current stage (51). Assessing GP's knowledge of patient's families may be applicable for more mature primary care practice in the future. Concerning comprehensiveness, nutrition guidance and health screening were selected. On one hand, the concept of medicine food homology is a traditional health-preserving philosophy with a long history in China (52). Nutrition counseling is also attracting increasing attention in GP's clinical practice (53, 54). On the other hand, health screening is an important preventive service provided for residents according to the National Basic Public Service Specifications issued by the Chinese government in 2017 (55).

RA-ASPC has advantages over other tools to evaluate the quality of primary care in China. Compared with other similar primary care assessment tools adapted in China, RA-ASPC could explain and capture more variance, indicating that it was necessary to develop new scales for practice in China. The total explained variance of RA-ASPC was 72.6%, higher than the modified PCAT adopted in different provinces in China, e.g., Guangdong (55.62%) (14), Changsha (56%) (11), and Tibetan (60%) (12); and the modified GPAQ adopted in Beijing (58.7%) (13). In addition, the Cronbach's alpha of RA-ASPC was also higher than that in previous validation studies, which ranged from 0.40 to 0.72 (14). Although internal consistency analysis showed good overall reliability of the RA-ASPC, three domains of the RA-ASPC have Spearman-Brown coefficients smaller than 0.7. This can be explained by the small number of items does not allow a comprehensive evaluation of each domain score, with such limitations also seen in other studies (56–58). The “Primary Care Assessment Tool (PCAT)” tool was previously abbreviated to a 10-item questionnaire by researchers for mass use in the national general health survey in Spain (56). Therefore, we also restricted the long ASPC to a 10-item scale to facilitate the future large-scale evaluation of primary care in China.

### Strengths and Limitations

There are several strengths of this study. First, in RA-ASPC, only 10 items were included in the scale, which would be much more time saving and feasible for daily management practice. Second, the study was conducted based on our previous work on developing and validating the original ASPC scale for assessing quality of primary care. The domains and the embedded definition in the scale were thoroughly considered based on the literature and also the current primary care practice in China. Third, the items selected to be included in RA-ASPC were evaluated by experts with rich experience in primary care after careful consideration of four aspects. Lastly, results of Cronbach's alpha, McDonald's Omega, EFA, CFA, and concurrent validity and discriminant validity supported the validity and reliability of RA-ASPC.

It is worth noting that although efforts have been made to present an accurate and comprehensive report in this study, the current study still suffers some limitations. Firstly, this study was conducted in one province, which may inevitably limit the generalizability of the results. However, we chose Guangdong province because the variations of geographical features and economic development levels between different regions in Guangdong province were similar to those between different provinces in China. Moreover, Guangdong province also has the largest population and the highest proportion of migrants in China. By collecting data in such a heterogeneous and large sample region, we expected to minimize the sample-specific bias and make it more representable to the current primary care practice in different provinces in China. When scaling up the survey to other provinces in China, it would be necessary to slightly adjust the wordings of RA-ASPC according to the language habits of the local population. The second limitation lies in the factor structure of the scale that a latent factor is explained by only two items. Although it is not very common, the previously theoretical structure of the scale and the sufficient statistical indexes could justify the final proposal of the RA-ASPC. The small number of items that contributed to the factors may lead to a low value of Cronbach's alpha and Spearman-Brown coefficients (58, 59). Lastly, the non-probability nature of purposive sampling may undermine the replicability of results generated. Nonetheless, we believe that the impact would be small as we have reported our sampling criteria and procedures transparently, which is relevant for replicability (60).

### Implication for Policy and Practice

The reliability and validity of RA-ASPC were established in our study. Specifically, RA-ASPC can be used to (i) monitor and evaluate the effectiveness of policies related to primary care (such as the Family Doctor Contract Services); (ii) provide valuable insights for performance evaluation between different regions and institutions *via* comparing the quality of primary care delivered by organizations and GPs; (iii) serve as an intermediate indicator between GP's health management and medical intervention and open the black box of service delivery process, thereby identify specific core functions for effective intervention.

## Conclusion

A 10-item RA-ASPC was developed and validated in the present study. The optimal psychometric properties of RA-ASPC indicated its appropriateness for assessing the quality of primary care from patient perspectives in China.

## Data Availability Statement

Data are available from the corresponding author on reasonable request.

## Ethics Statement

Ethical approval was obtained from the Institutional Review Board of School of Public Health, Sun Yat-sen University, China (2018.014). The protocol for this study conforms to the principles embodied in the Declaration of Helsinki.

## Author Contributions

LK: study design. CZ, LL, ZL, CL, and MZ: data acquisition. CZ, JH, LL, and LK: data interpretation. CZ: tables, figures, and appendix preparation. CZ and LK: drafting manuscript. CZ, JH, and LK: critical revision of the manuscript. All authors have read and approved the manuscript.

## Funding

This study was supported by the National Natural Science Foundation of China (Grant No: 71673311). The funder does not interfere in our research. The funding body was not involved in the design of the study; data collection, analysis, and interpretation; or writing the manuscript. The process was all completed by our research group independently.

## Conflict of Interest

The authors declare that the research was conducted in the absence of any commercial or financial relationships that could be construed as a potential conflict of interest.

## Publisher's Note

All claims expressed in this article are solely those of the authors and do not necessarily represent those of their affiliated organizations, or those of the publisher, the editors and the reviewers. Any product that may be evaluated in this article, or claim that may be made by its manufacturer, is not guaranteed or endorsed by the publisher.
