# Supplementary material for: Development and Validation of a Rapid Assessment Version of the Assessment Survey of Primary Care in China
Source: Front Public Health. 2022 May 10;10:852730. doi: 10.3389/fpubh.2022.852730 (PMC9127135; doi:10.3389/fpubh.2022.852730)
Supplement: Supplementary file 1 [file Data_Sheet_1.pdf]

Appendix 1. Experts perceived importance, representativeness, easy-understanding, and general applicability of each item in the five domains of the original ASPC scale

a) Experts perceived importance, representativeness, easy-understanding, and general applicability of each item in Domain 1: First-contact care

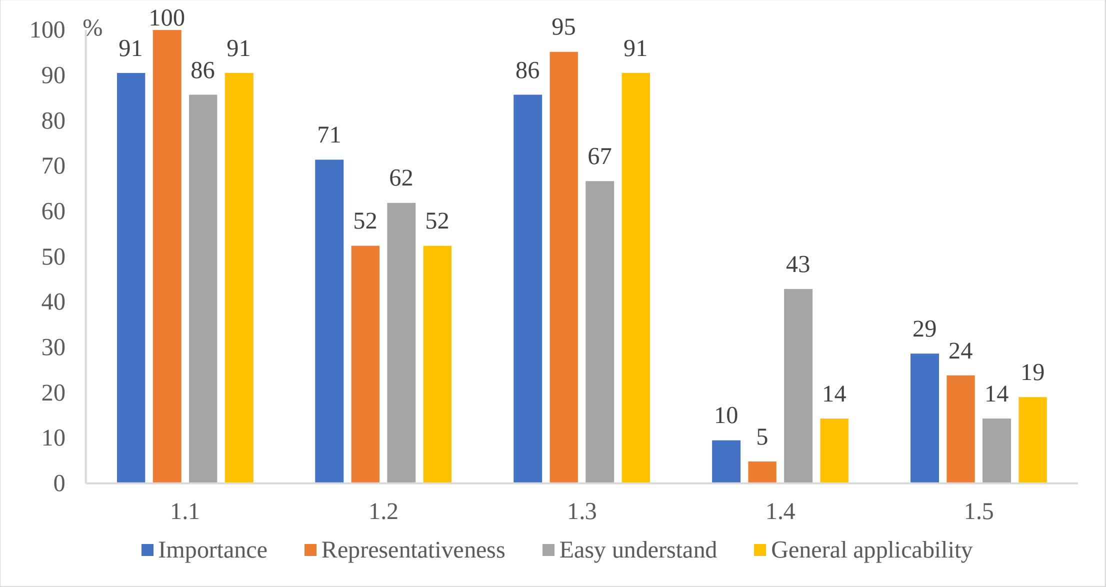

Notes:

Item 1.1: When you felt unwell (e.g., get a cold, cough, fever, etc.), did you go to see the general practitioner in the first instance?

Item 1.2: When you experienced flare-ups of your chronic diseases, did you go to see the general practitioner in the first instance?

Item 1.3: When you need health counseling, did you go to see the general practitioner in the first instance?

Item 1.4: When you need health check-up or health examination, did you go to see the general practitioner in the first instance?

Item 1.5: When you need preventive care, did you go to see the general practitioner in the first instance?

b) Experts perceived importance, representativeness, easy-understanding, and general applicability of each item in Domain 2: Continuity

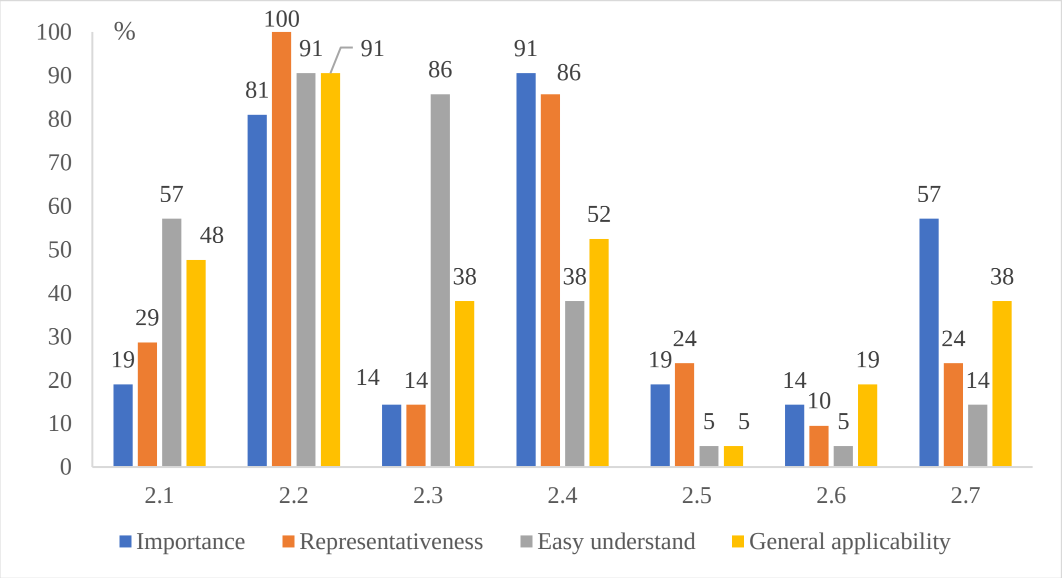

Notes:

Item 2.1: How many years have you been seeing the general practitioner?

Item 2.2: Did you often see the same general practitioner when you went to the primary care setting for counseling?

Item 2.3: Did you often see the same general practitioner when you went to the primary care setting for prescription?

Item 2.4: Did the general practitioner know about your medical history comprehensively?

Item 2.5: Did the general practitioner know your family member?

Item 2.6: Did the general practitioner know about your family members' medical history?

Item 2.7: Did the general practitioner take the initiative to schedule follow-up with you?

c) Experts perceived importance, representativeness, easy-understanding, and general applicability of each item in Domain 3: Accessibility

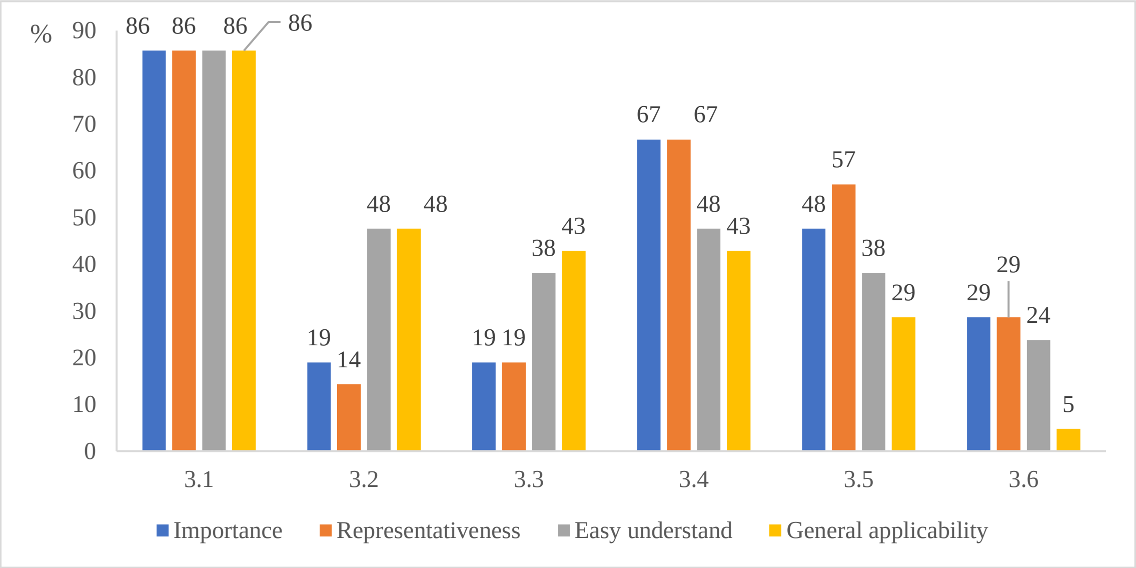

Notes:

Item 3.1: If necessary, could you see the general practitioner at office hour?

Item 3.2: How long did you wait outside the consultation room before you can see the general practitioner?

Item 3.3: Did you feel it a long time to wait outside the consultation room?

Item 3.4: If necessary, could you see the general practitioner at night?

Item 3.5: If necessary, could you see the general practitioner at weekend?

Item 3.6: If necessary, could you see the general practitioner at home?

d) Experts perceived importance, representativeness, easy-understanding, and general applicability of each item in Domain 4: Comprehensiveness

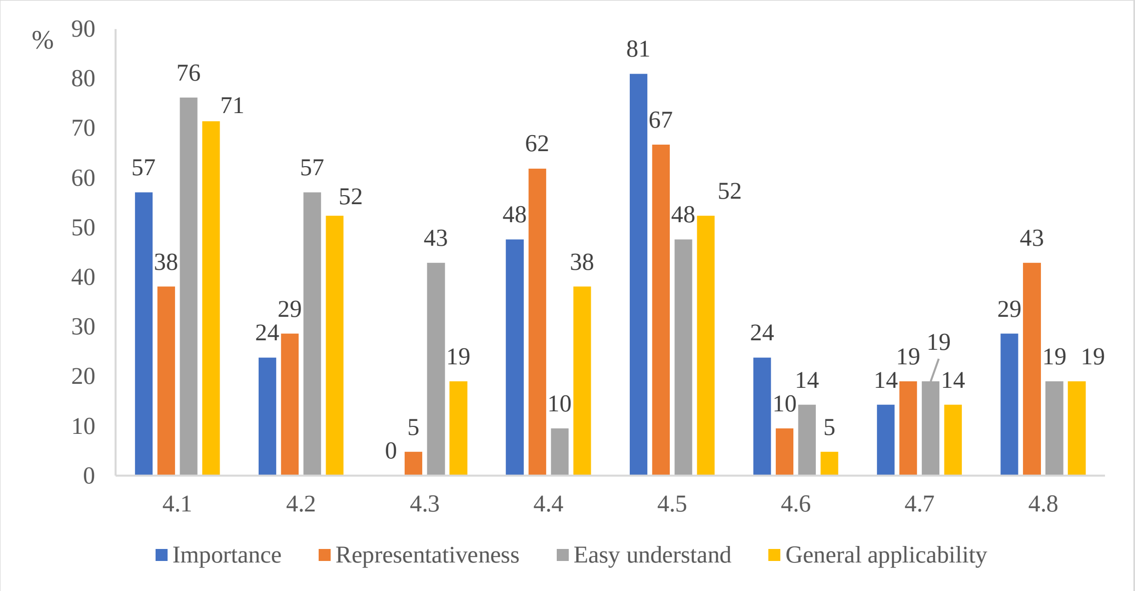

Notes:

Item 4.1: Did you often receive tailored nutrition advice from the general practitioner?

Item 4.2: Did you often receive tailored exercise advice from the general practitioner?

Item 4.3: Did you often receive tailored smoking advice from the general practitioner?

Item 4.4: Did you often receive tailored psychosocial support from the general practitioner?

Item 4.5: Did you often receive tailored health screening advice from the general practitioner?

Item 4.6: Did you often receive tailored health screening advice from the general practitioner?

Item 4.7: Did you often receive tailored vaccination advice from the general practitioner?

Item 4.8: Did you often receive community-based health advice from the general practitioner?

e) Experts perceived importance, representativeness, easy-understanding, and general applicability of each item in Domain 5: Coordination

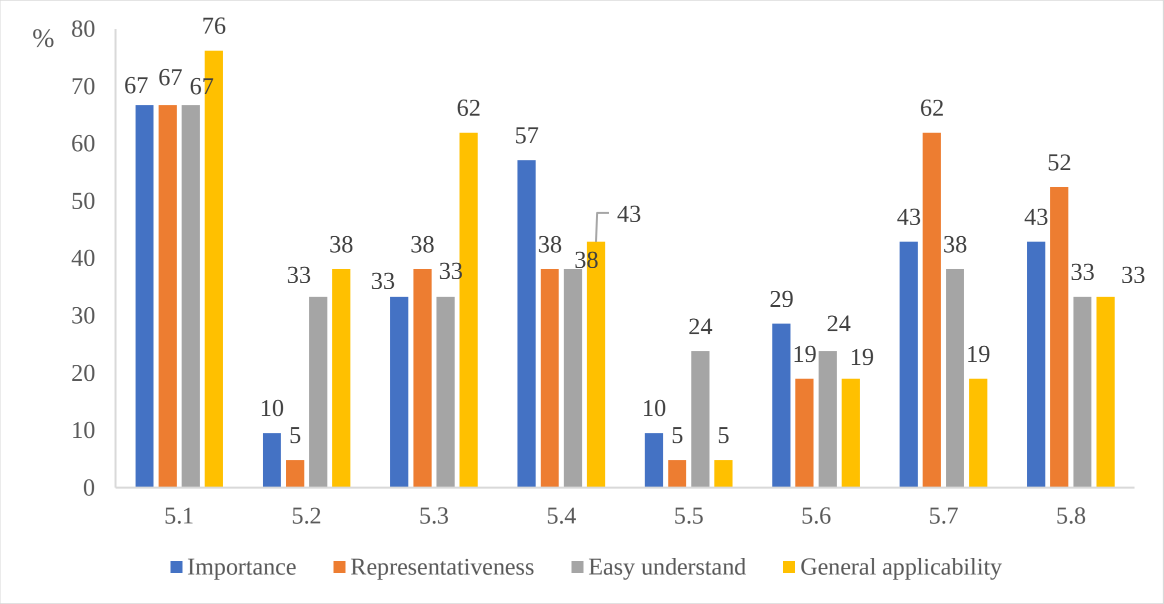

Notes:

Item 5.1: Did you often consult your general practitioner if you need to transfer to the hospital?

Item 5.2: Did you often inform your general practitioner about your previous treatment plan in the hospital?

Item 5.3: Did your general practitioner often take the initiative to ask about your previous treatment plan in the hospital?

Item 5.4: Did your general practitioner often discuss with you about the reason of your transferal?

Item 5.5: Did your general practitioner often discuss with you about which hospital to transfer?

Item 5.6: Did your general practitioner often discuss with you about which department to transfer?

Item 5.7: Did your general practitioner often contact the hospital for you to transfer?

Item 5.8: Did your general practitioner often provide your complete medical record in the transferal letter for you?

Appendix 2. Details of each item constituted the five domains of RA-ASPC[1]

Domains	Definition of each domain	Items included in each domain
First-contact care	Patient's experience of primary care as the first point of contact for the majority health needs	Item 1.1: When you felt unwell (e.g., get a cold, cough, fever, etc.), did you go to see the general practitioners in the first instance?
		Item 1.3: When you need health counseling, did you go to see the general practitioners in the first instance?
Continuity	The interpersonal relationship between the general practitioner and the patient	Item 2.2: Did you often see the same general practitioner when you went to the primary care setting for services?
		Item 2.4: Did the general practitioner know about your medical history comprehensively?
Accessibility	The accessibility of primary care services provided by primary health care institutions and general practitioners	Item 3.1: If necessary, could you see the general practitioner at office hour?
		Item 3.4: If necessary, could you see the general practitioner at night?
Comprehensiveness	The range of all types of primary care services including clinical medical services and preventive services provided to patients	Item 4.1: Did you often receive tailored nutrition advice from the general practitioner?
		Item 4.5: Did you often receive tailored health screening advice from the general practitioner?
Coordination	Coordinated services provided by general practitioners before and after patient's transferal to hospital/specialist	Item 5.1: Did you often consult your general practitioner if you need to transfer to the hospital?
		Item 5.4: Did your general practitioner often discuss with you about the reason of your transferal?

Note: RA-ASPC: Rapid assessment of the ASPC scale

Reference:

1. Kuang L, LL, Luo Z, Zhong C, Liang C, Zhou M. Development and Validation of the Assessment Survey of Primary Care (ASPC) in China. Chinese General Practice. 2021;24(13):1621-8.

Appendix 3. Relationship between the total score and score in each domain of RA-ASPC and patient satisfaction with the general practitioner

Domains	Patient satisfaction				
	β	95%CI		*SE*	*P*
First-contact care	2.89	2.03	5.60	0.439	<0.001
Continuity	5.10	4.19	6.02	0.466	<0.001
Accessibility	2.53	1.17	3.90	0.697	<0.001
Comprehensiveness	3.67	2.88	4.47	0.404	<0.001
Coordination	4.54	3.48	5.60	0.540	<0.001
Total score	10.2	8.68	11.7	0.369	<0.001

Notes: RA-ASPC: Rapid assessment of the ASPC scale. Linear regression model was adopted to analysis the relationship between the total score and score in each domain of RA-ASPC and patient satisfaction with the general practitioner, after controlling for patient's gender, age, marital status, education level, monthly household income, work status, household status, health insurance, health status, chronic disease, number of years since first visit to the primary care setting, whether have a family doctor or not.

Appendix 4. Total score and score in each domain of RA-ASPC between patients with or without a family doctor

Domains	Without family doctors		With family doctors		*Diff (I-II)*	*P*
	Mean (I)	SE	Mean (II)	SE		
First-contact care	3.34	0.031	3.52	0.042	-0.175	0.002
Continuity	2.59	0.028	2.99	0.038	-0.391	<0.001
Accessibility	3.54	0.020	3.58	0.027	-0.040	0.254
Comprehensiveness	2.67	0.034	3.00	0.045	-0.329	<0.001
Coordination	2.63	0.025	2.79	0.034	-0.152	0.001
Total score	2.96	0.027	3.17	0.023	-0.217	<0.001

Notes: RA-ASPC: Rapid assessment of the ASPC scale. SE standard error. *Diff*: absolute difference between the two groups.
* Multivariate analyses of covariance controlling for patient's gender, age, marital status, education level, monthly household income, work status, household status, health insurance, health status, chronic disease, number of years since first visit to the primary care setting.
